# 3D DESI-MS lipid imaging in a xenograft model of glioblastoma: a proof of principle

**DOI:** 10.1038/s41598-020-73518-x

**Published:** 2020-10-05

**Authors:** Fiona Henderson, Emrys Jones, Joanna Denbigh, Lidan Christie, Richard Chapman, Emmy Hoyes, Emmanuelle Claude, Kaye J. Williams, Federico Roncaroli, Adam McMahon

**Affiliations:** 1 Wolfson Molecular Imaging Centre, Division of Informatics, Imaging and Data Sciences, School of Health Sciences, Faculty of Biology, Medicine and Health, University of Manchester, Manchester Academic Health Science Centre, Manchester, M20 3LJ UK; 2 Division of Pharmacy and Optometry, School of Health Sciences, Faculty of Biology, Medicine and Health, University of Manchester, Manchester Academic Health Science Centre, Stopford Building, Manchester, M13 9PT UK; 3 Waters Corporation, Wilmslow, SK9 4AX UK; 4 University of Salford, Salford, Manchester, UK; 5 Division of Neuroscience and Experimental Psychology, Faculty of Biology, Medicine and Health, University of Manchester and Manchester Centre for Clinical Neuroscience, Salford, UK

**Keywords:** Biochemistry, Biological techniques, Cancer, Biomarkers, Diseases, Medical research, Oncology

## Abstract

Desorption electrospray ionisation mass spectrometry (DESI-MS) can image hundreds of molecules in a 2D tissue section, making it an ideal tool for mapping tumour heterogeneity. Tumour lipid metabolism has gained increasing attention over the past decade; and here, lipid heterogeneity has been visualised in a glioblastoma xenograft tumour using 3D DESI-MS imaging. The use of an automatic slide loader automates 3D imaging for high sample-throughput. Glioblastomas are highly aggressive primary brain tumours, which display heterogeneous characteristics and are resistant to chemotherapy and radiotherapy. It is therefore important to understand biochemical contributions to their heterogeneity, which may be contributing to treatment resistance. Adjacent sections to those used for DESI-MS imaging were used for H&E staining and immunofluorescence to identify different histological regions, and areas of hypoxia. Comparing DESI-MS imaging with biological staining allowed association of different lipid species with hypoxic and viable tissue within the tumour, and hence mapping of molecularly different tumour regions in 3D space. This work highlights that lipids are playing an important role in the heterogeneity of this xenograft tumour model, and DESI-MS imaging can be used for lipid 3D imaging in an automated fashion to reveal heterogeneity, which is not apparent in H&E stains alone.

## Introduction

The revised 4th edition of the classification edited by the World Health Organisation (WHO) recognises more than 120 tumour entities with distinctive morphology, location, age distribution and biologic behaviour. Tumour entities are graded with a ‘malignancy scale’ that is intended to predict their natural biological behaviour. Gliomas account for the commonest primary brain tumours in adults. They are graded from I to IV based on histological features. The identification of molecular and genetic signatures such as mutations in the isocitrate dehydrogenase 1 and 2 (IDH1/2) genes, ATRX and hTERT, CDKN2A/B deletions, EGFR gene amplifications and co-deletions of chromosomes 1p19q has improved the diagnostic accuracy and stratification of patients with glioma. Glioblastoma (GBM) (WHO grade IV) is the most aggressive among gliomas with a patients’ mean survival of 15 months from onset^1^. GBM can occur as a primary tumour or can result from the progression of a lower grade astrocytoma. Primary (de novo) GBM is the most common type accounting approximately for 90% of cases. Glioblastomas are highly heterogeneous tumours. Regional genetic, epigenetic and metabolic differences determine intratumoural heterogeneity. Intratumour heterogeneity is a result of evolutionary changes that occur following dynamic remodelling of the tumour microenvironment and the effects of treatment^2,3^. These genetic alterations can contribute towards resistance to therapy^4^. Among metabolic changes in GBM, lipid levels including free fatty acids are altered compared to normal tissue^5,6^ as the result of hypoperfusion and subsequent hypoxia^7,8^. Fatty acid synthase (FASN) is responsible for de novo synthesis of fatty acids, and is upregulated in gliomas^9^, amongst other cancer types^10,11^, with an increase in expression being associated with malignant gliomas^12^.

Gliomas are diagnosed and monitored with magnetic resonance imaging (MRI). Molecular imaging modalities such as positron emission tomography (PET) can also contribute to the management of patients with glioma. Both MRI and PET have progressed substantially in investigating the heterogeneity of glial tumours as they can image whole tumours in-vivo^13–15^ but they may fail to detect subtle but relevant structural or functional features due to their low spatial resolution.

Mass spectrometry imaging is an *ex-vivo* molecular imaging method that offers advantages over PET and MRI because it can image hundreds of molecules at a spatial resolution below 200 μm. Desorption electrospray ionisation mass spectrometry (DESI-MS) is an ambient mass spectrometry technique which has been used for molecular imaging since 2004^16–18^. Although DESI-MS is an *ex-vivo* technique, it causes minimal degradation to the sample, and so staining is possible on the same section post-DESI-MS imaging, making it an ideal tool for imaging brain tumours. Unlike *in-vivo* techniques such as PET or MRI, MS imaging only captures 2D images. However, MS imaging can acquire serial tissue sections, and stack as 3D images. Matrix-assisted laser desorption ionisation (MALDI-MS) imaging is the most commonly used MS imaging technique and has already been used for 3D imaging^19^ including the mapping of drug distribution in tumours^20^. Eberlin et al*.* reported 3D DESI-MS imaging of a mouse brain^21^. 3D MS imaging is a lengthy process and involves human input to set up and acquire new images. Here, for the first time, an automated 3D DESI-MS imaging method has been used to visualise tumour heterogeneity.

A robotic slide loader that can autonomously mount and change slides on the DESI-MS instrument allows rapid acquisition of a set of 2D images that are automatically processed and aligned to render a 3D image. This enables straightforward and rapid generation of 3D DESI-MS images. DESI-MS is an excellent tool for the detection of lipids, and has been used in numerous lipidomic studies in cancer^22–24^. In gliomas, DESI-MS lipid fingerprints could distinguish between healthy and tumour tissue and between different glioma grades^25,26^.

In this study we performed 3D DESI-MS imaging using a heterotopic xenograft glioblastoma model to investigate lipid heterogeneity and immunofluorescence to find correlations between the MS lipid heterogeneity data and the downstream marker of hypoxic signalling, carbonic anhydrase-9 (CA-9).

## Results and discussion

**Figure 1:**
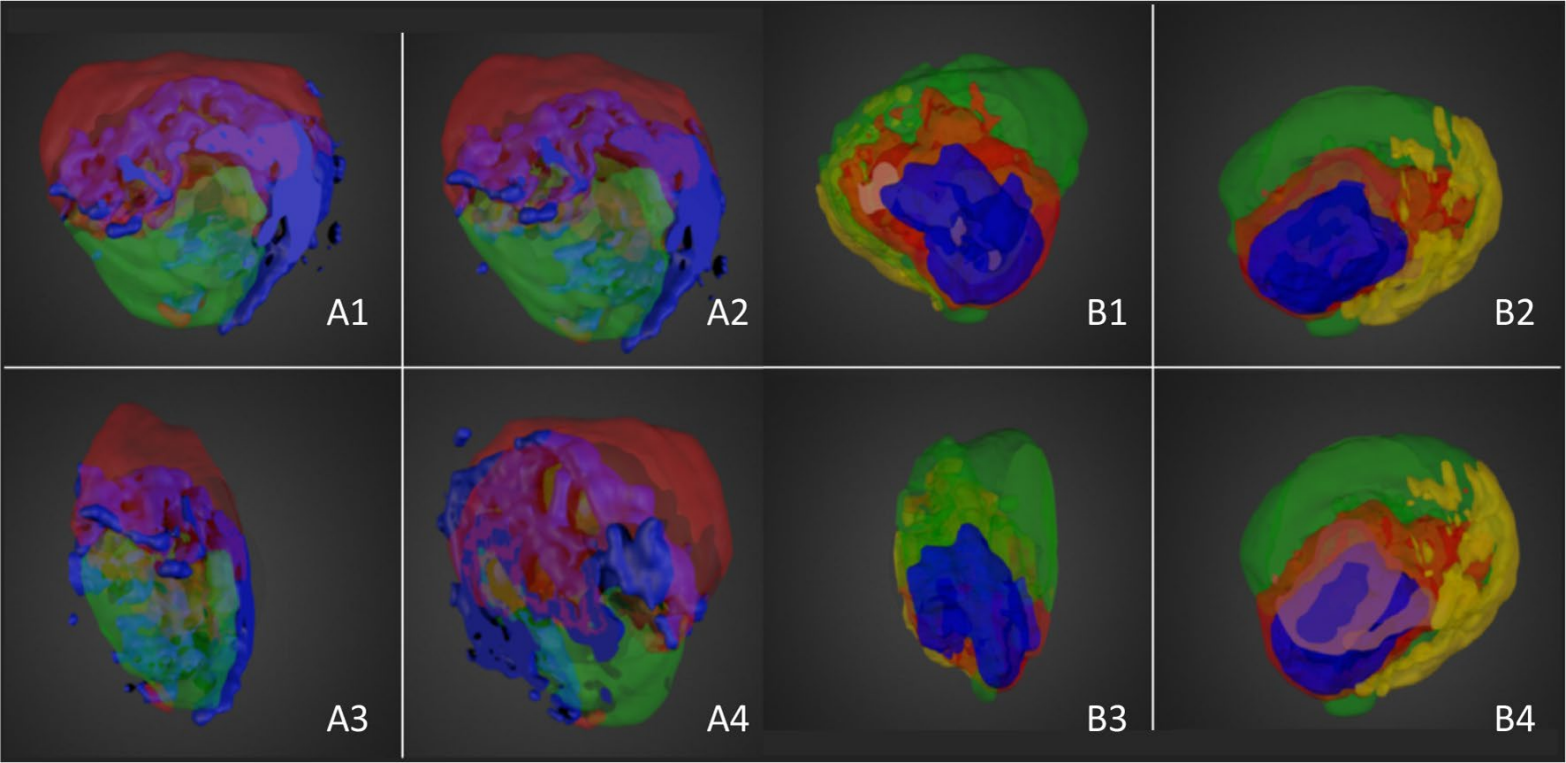
3D DESI-MS images of molecularly distinct regions (displayed in different colours) as revealed by multivariate analysis. Each frame shows a different 2D perspective as the 3D image is rotated. A1–A4: positive ion images, B1–B4: negative ion images.

Fifteen sections were taken every 120 μm from each GBM xenograft and underwent DESI-MS imaging in both negative and positive ion mode. The 2D data were combined and reconstructed to generate 3D DESI-MS images for both polarities. Multivariate analysis (MVA) was performed on the 3D data set to reveal areas of the tumour which had different regions according to their lipid profiles (Fig. 1). 3D DESI-MS imaging was therefore able to visualise areas of distinct lipid heterogeneity within the tumour.

**Figure 2:**
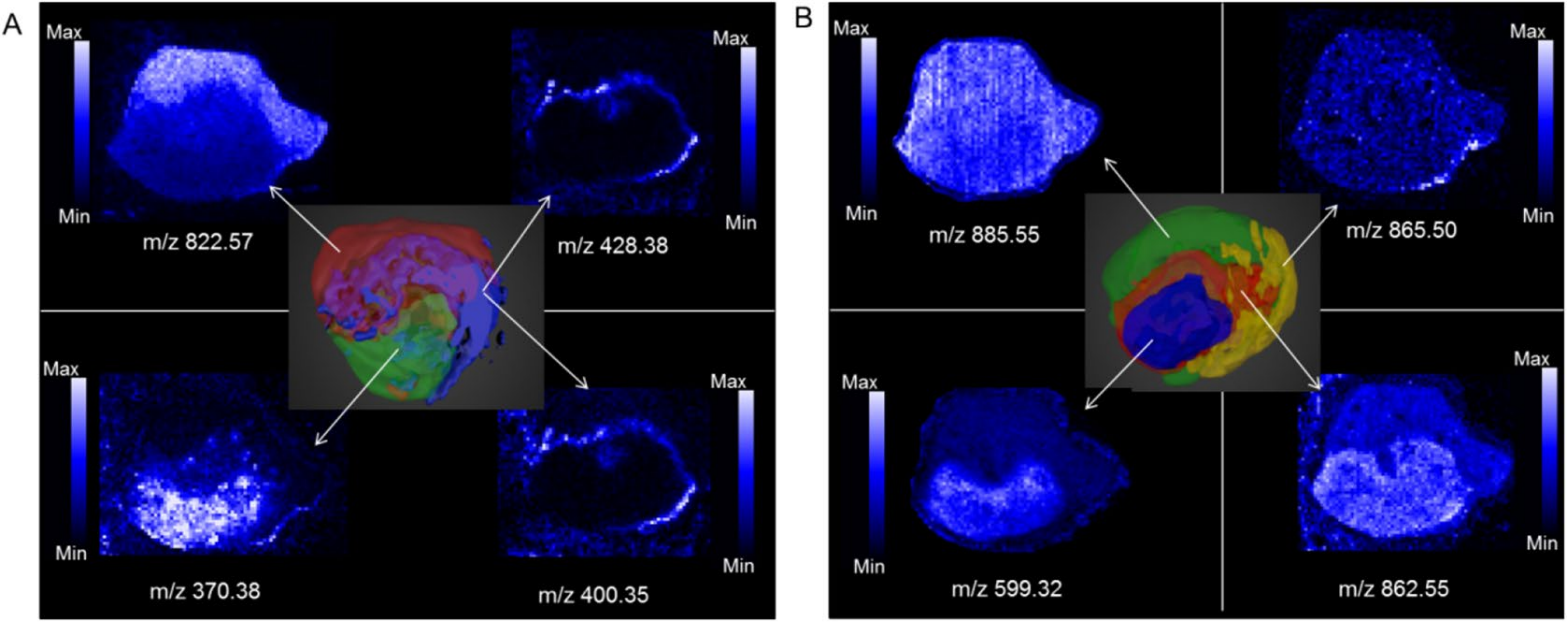
DESI-MS images in positive (**A**) and negative (**B**) ion mode of peaks reflecting molecularly different regions.

2D images of representative peaks from the distinct tumour areas as separated by the MVA is shown in Fig. 2. A peak appearing in negative ion mode at m/z 885 has been tentatively identified as PI (38:4) based on exact mass and what has previously been reported in the literature, with Eberlin et al*.* also reporting m/z 885 (PI 38:4) in viable GBM^25^. Sections adjacent to those used for DESI-MS were stained with CA-9 (to visualise hypoxic regions) and H&E, these were interpreted by a neuropathologist. Molecularly different regions visualised in the DESI-MS images showed different cellularity, with hypoxic regions appearing less cellular than the remaining tumour.

**Figure 3:**
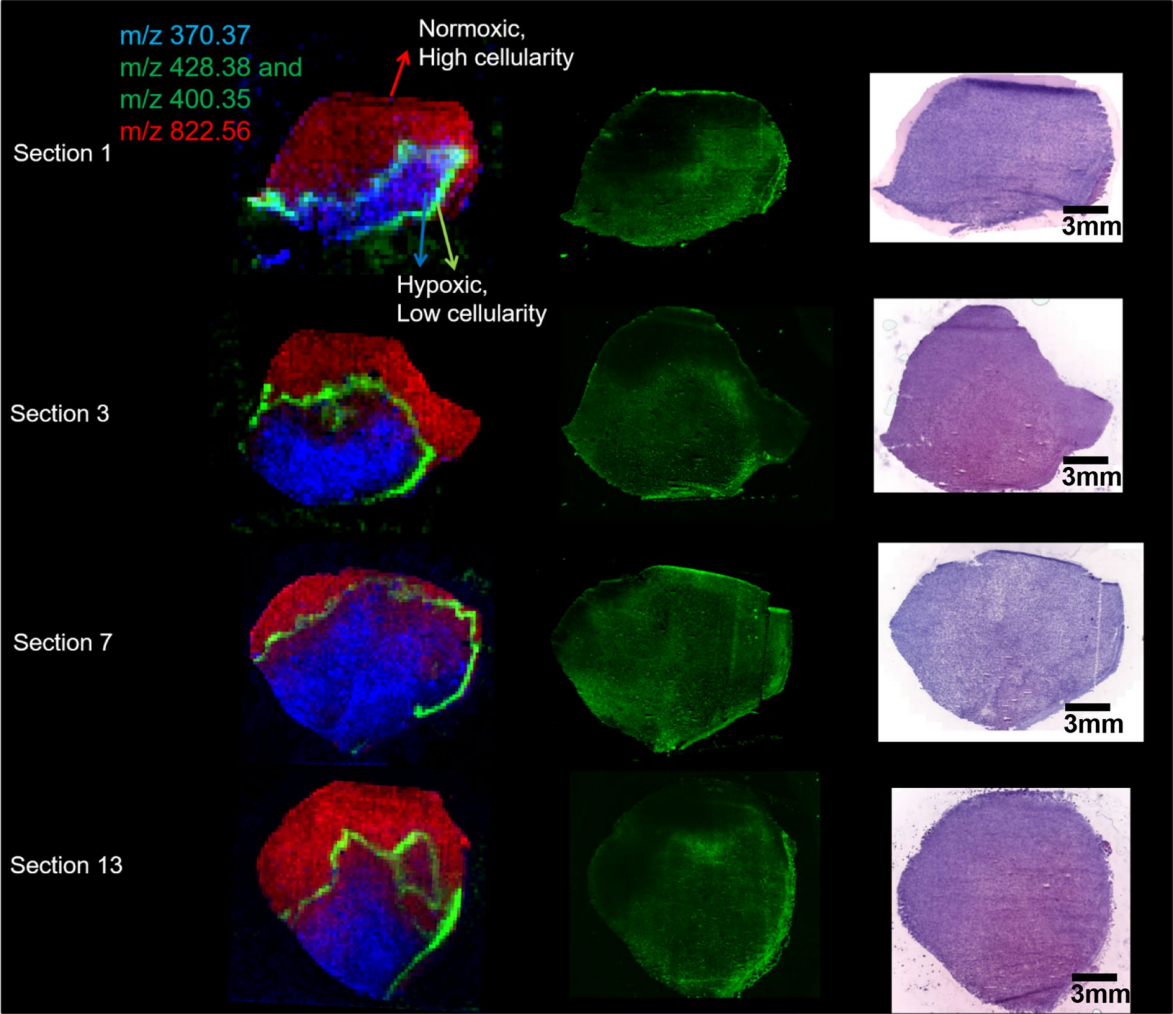
2D DESI-MS images in positive ion mode showing lipids that localise in different regions of the tumour (left), with corresponding CA-9 stains (middle), and H&E images (right) for different sections of the GBM.

**Figure 4:**
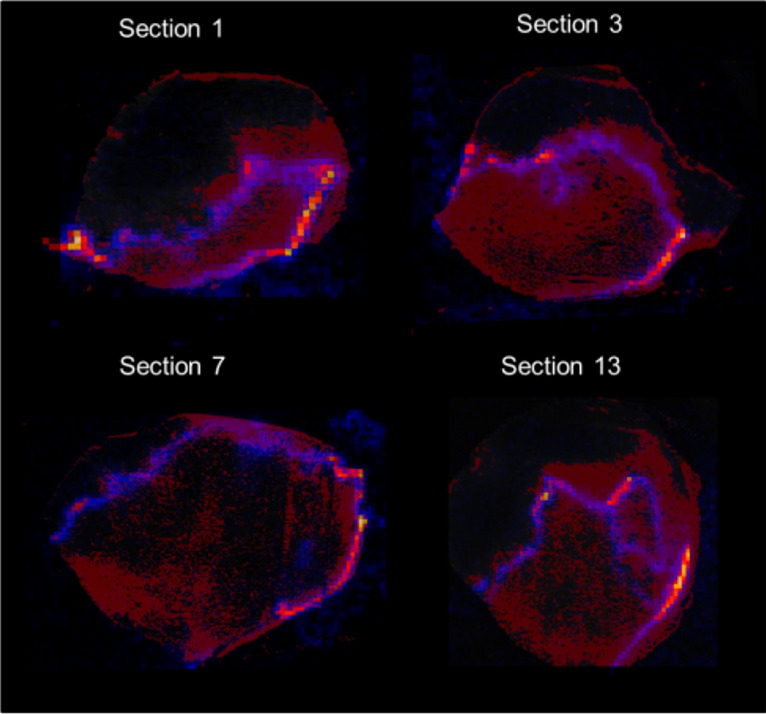
2D positive ion mode DESI-MS images, overlaid with CA-9 staining for different sections of the GBM.

**Figure 5:**
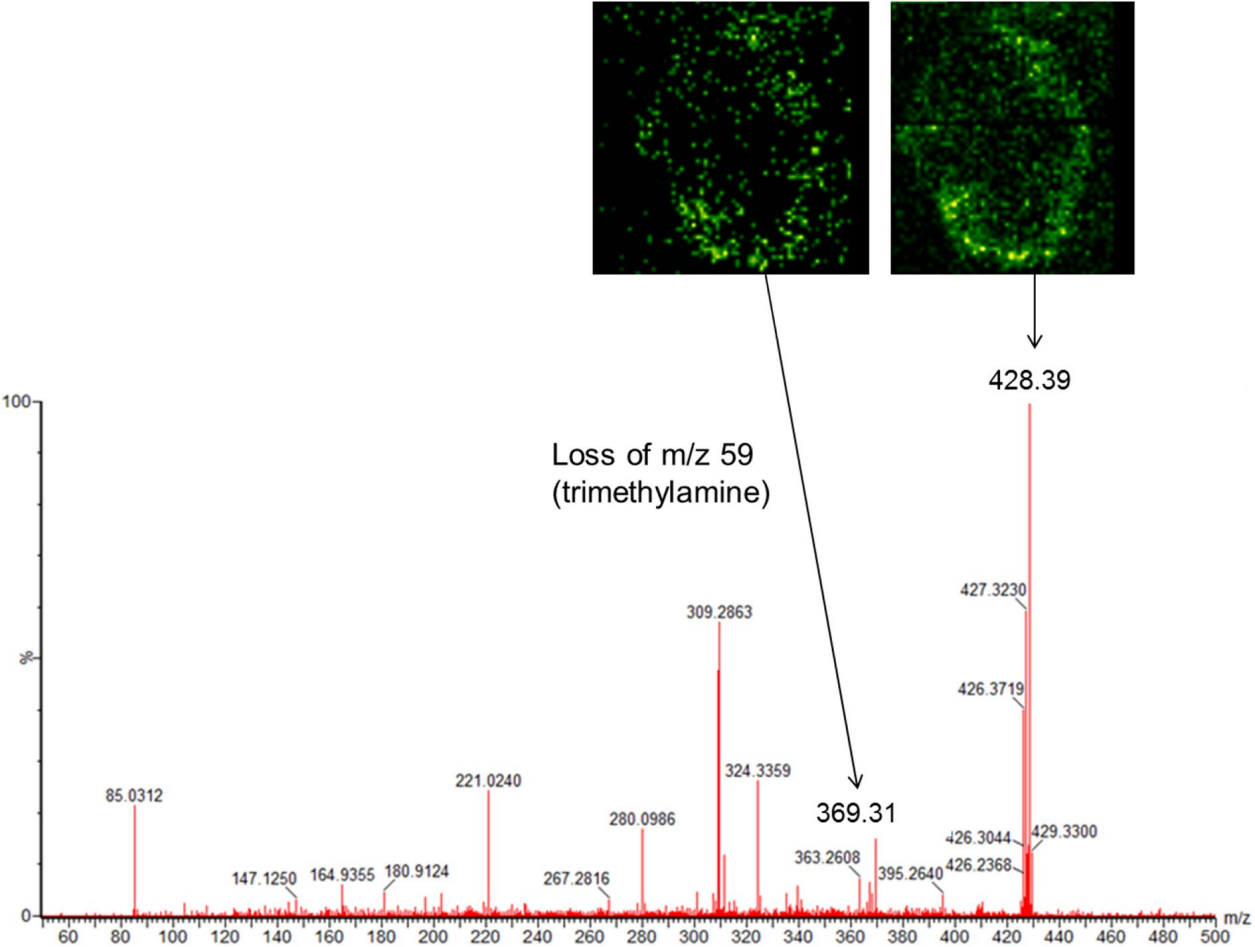
Positive DESI-MS/MS of m/z 428 averaged across the image, including images of the parent and m/z 369 fragment ion.

Comparing DESI-MS images with CA-9 staining (Figs. 3 and 4) revealed that the peaks at m/z 400 and m/z 428 (positive ion mode) co-localise with areas of lower cellularity (shown as blue in Fig. 3). The peaks at m/z 400 and m/z 428 co-localised with hypoxia but only in some hypoxic regions forming a thin band within the areas of CA-9 staining. The m/z 400 and 428 band was overlaid with CA-9 images to visualise exactly where in the hypoxic area the m/z 400/428 band was forming (Fig. 5). The m/z 400/428 band was found to localise in the midst of CA-9 staining; rather than forming a boundary between hypoxic and normoxic tissue. These findings suggest that the lipids at m/z 400 and 428 may only be upregulated in very specific oxygen tensions. Furthermore, the 3D positive ion mode DESI-MS image was able to visualise this metabolically unique area, allowing three dimensional interrogation of this heterogeneity.

Peaks observed at m/z 428 and m/z 400 have been identified as stearoylcarnitine and palmitoylcarnitine respectively, based on the Lipid Maps database^27^ and what has been previously reported in hypoxic areas^28^. In addition, the MS/MS spectrum for m/z 428 obtained from the tissue samples (Fig. 5), shows a fragment peak at 369, representative of the loss of a trimethylamine group from the carnitine. The MS/MS imaging shows that this fragment co-localises with the m/z 428 parent peak (Fig. 5, inset images).

Acyl-carnitines act as intermediates to shuttle fatty acids into the mitochondria. Long chain fatty acids cannot cross the mitochondrial membranes for reasons of permeability and must be converted to fatty acyl-CoAs, which are in turn converted to acylcarnitines. In the mitochondria, acylcarnitines are converted back to fatty acyl-CoAs to undergo β-oxidation and eventually produce energy. The import into the mitochondria is the rate limiting step of fatty acid oxidation and is carried out by various carnitine transferases.

Lin et al*.* reported that fatty acid oxidation is prevalent in cells derived from human gliomas and that it plays an important role in proliferation. They also found that inhibition of fatty acid oxidation leads to tumour growth and increased survival in a syngeneic mouse glioma model^29^. This finding concurs with the work of Huang et al*.,* who demonstrated the importance of the hypoxia inducible factor-1α (HIF-1α) protein in inhibiting fatty acid oxidation at low oxygen tensions, resulting in cancer cell proliferation^30^. Interestingly, we report an increase in β-oxidation within a subset of tumour hypoxia (relative to the normoxic regions of the U87 xenograft model), eluding to complex mechanisms at play within specific oxygen tensions.

Zaugg et al*.* discussed the role of carnitine palmitoyltransferase 1C (CPT1C), a brain-specific enzyme responsible for transporting carnitines across the mitochondrial membrane. Their findings suggest that hypoxia can cause upregulation of CPT 1C in vivo^31^*,* in keeping with our evidence of an increase in palmitoylcarnitine in hypoxic regions of the U87 xenograft tumour model. These findings support the complex role of hypoxic regulation of carnitines in GBM, where their upregulation at specific oxygen tensions confers a metabolic advantage to hypoxic cells.

## Conclusion

In conclusion, we have devised a method for 3D DESI-MS imaging of a glioma model and analysed its heterogeneity. Automation of experiment and of 3D visualisation facilitates its use as a high throughput imaging modality that is suitable for large cohorts of samples. Furthermore, the use of consecutive sections allows the acquisition of a considerable amount of information on the biology of the tumour analysed. Despite the limitations of our U87 xenograft model, distinct regions of heterogeneous lipid expression that correlate with the varying levels of local oxygen tension and palmitoyl- and stearoyl-carnitines, appear to be playing key roles to aid β-oxidation in hypoxic areas of GBMs. Mapping on tissue the metabolic shift to lipid metabolism in regions with low oxygen tension is relevant to better understanding tumour metabolic heterogeneity in GBM and the impact of hypoxia on the dynamic changes of tumour microenvironment and its consequences on resistance to radiation treatment and chemotherapy^32–35^.

## Methods

### Materials

Phosphate buffered saline (PBS) was made using tablets purchased from Oxoid, UK, and dissolved in dH_2_O.

1 ml Tween (T) was added to PBS to make PBS-T. BSA (Sigma-Aldrich, UK) was dissolved at 1 mg/ml PBST to make PBST-BSA solution.

All solvents were purchased from Sigma-Aldrich, UK.

### In-vivo experiments

Animal experiments were conducted in accordance with the U.K. Animals (Scientific Procedures) Act 1986, and had institutional and regulatory ethical approval (University of Manchester Animal Welfare and Ethical Review- AWERB) under Home Office license PPL707760 (granted to KJW). 5 × 10^6^ U87 cells were injected intradermally into the flank of a female cba nu/nu mice (age 10–14 weeks/weight 20–24 g). Mice were maintained under dawn-til-dusk 12 h light/dark cycles at an average ambient temperature of 21 °C (range 19–23 °C), relative humidity average 55% (range 45–65%) in pathogen free housing (Techniplast GM500 Mouse IVC Green Line) with sterilized bedding, environmental enrichment and access to food water ad libitum with water supplied by Hydropac watering system. Weights, body condition and behaviour were monitored routinely throughout the experiments as measures of animal well-being. After 4 weeks animals were culled and tumours flash frozen in isopentane.

### Sample preparation

A xenograft glioblastoma tumour was sectioned using a cryostat (Leica 3050 s, UK), and tissue sections were thaw mounted onto glass slides. Fifteen sections were taken at 120 μm steps through the tumour, at a 10 μm thickness for DESI-MS imaging. Serial sections (8 μm) were taken for immunofluorescence.

### DESI-MS 3D imaging

Experiments were carried out using a 2D DESI stage (Prosolia, Indianapolis,USA) attached to a Xevo-G2-XS quadrupole- time of flight (Q-TOF) (Waters, UK). A modified top plate of the DESI stage (Prior Scientific, Cambridge) was created to allow the automated PL200 microscope slide loader (Prior Scientific, Cambridge) to place slides onto the stage, and then move them back to the holding cassette. A snapshot of the slide and object definition algorithms were used to identify the location of the tissue(s) on the slide. These co-ordinates were then written to the experiment file and imaging commenced. This process continued until all 15 slides were analysed. Spatial resolution was set to 120 μm, in order to make even sized voxels. A mass range of m/z 50–1200 was used. Spray conditions used were 5 µl/min, 95% methanol 5% water, with a nebulising gas of nitrogen at 6 bar pressure. Typical positions of the sprayer were used (sprayer 1.5 mm above surface, 6 mm sprayer to capillary distance, 75° sprayer impact angle, 5° collection capillary angle). Imaging was carried out in both negative and positive ion mode, at 10 scans/sec. The capillary voltage was 2.3 kV in negative ion mode, and 3.42 kV in positive ion mode.

HDImaging v1.4 (Waters, Milford, USA) was used for visualisation of individual 2D images, where images were normalised to the total ion current (TIC). Recently developed software from Waters, UK allowed reconstruction of the sections into a 3D image of the molecularly distinct regions within the whole tissue volume. The reconstruction was carried out automatically, and involved peak picking, object alignment and rotation, as well as a non-negative matrix factorisation (NNMF).

### DESI-MS/MS

Conditions were the same as DESI-MS imaging. MS/MS was carried out using 7 bar of argon gas. Lipidomics Gateway database https://www.lipidmaps.org^27^ was used for identification of species.

### Immunofluorescence

Tissue sections were fixed in ice-cold acetone for 10 min and then left to dry. A DAKO pen (Agilent Technologies, UK) was used to draw around each of the sections, and 70 μl of blocking solution was pipetted onto each section (10% goat serum in PBST), and incubated at room temperature for 15 min. Slides were then washed twice for 3 min in PBST-BSA. Anti-CA-9 (mouse, Biosciences, Slovakia) at 1:1000 was made up in PBST-BSA, and 10% normal goat serum. ~ 70 μl of primary antibody solution was left on tissue sections at 4 °C overnight. The primary antibody solution was tapped off and the sections washed three times for four mins in PBS. A secondary antibody solution containing 1:100 Alexafluor 488 goat anti-mouse (Life technologies, UK) was prepared in PBST-BSA; and 70 μl was added to each section and left for 60 min in the dark at room temperature. Slides were washed again with PBS (3 × 4 min). Coverslips (VWR, UK) were then mounted using fluorescent mounting media (Abcam, UK). Images were acquired using a [*20x/0.80 Plan Apo*] objective using the 3D Histech Pannoramic 250 Flash II slide scanner, and processed using Panoramic viewer (3D HISTECH, Hungary). Images were also processed in Image J^36^, where images were converted to a 32-bit greyscale image, and a default threshold applied.

### H&E

Sections adjacent to those used for DESI-MS were stained using haematoxylin and eosin (H&E). Following submersion in xylene (Sigma-Aldrich, UK) for 2 min, slides were immersed 4 times for 2 min in industrial methylated spirit (Sigma-Aldrich, UK). Following washing in tap water, sections were stained using Harris haematoxylin (3 min; Sigma-Aldrich, UK), rinsed in hot water, and dipped for one second in eosin (Leica, UK). Following further industrial methylated spirit (4 × 2 min) and xylene (4 × 2 min) washes, slides were left to dry. Samples were mounted with coverslips using aqueous mounting media (Abcam, UK) and left overnight to dry. Images were acquired using the 3D Histech Pannoramic 250 Flash II slide scanner with [*20x/0.80 Plan Apo*] objective and processed using Pannoramic viewer (3D HISTECH, Hungary).

## Data Availability

The datasets generated during and/or analysed during the current study are not publicly available as they are still being mined with the hope of a future publications, but are available from the corresponding author on reasonable request.
